# Probiotic treatment reduces appetite and glucose level in the zebrafish model

**DOI:** 10.1038/srep18061

**Published:** 2016-01-05

**Authors:** Silvia Falcinelli, Ana Rodiles, Suraj Unniappan, Simona Picchietti, Giorgia Gioacchini, Daniel Lee Merrifield, Oliana Carnevali

**Affiliations:** 1 Dipartimento di Scienze della Vita e dell’Ambiente, Università Politecnica delle Marche, Ancona, Italy.; 2 Aquatic Animal Nutrition and Health Research Group, School of Biological Sciences, Plymouth University, PL4 8AA, UK.; 3 Laboratory of Integrative Neuroendocrinology, Department of Veterinary Biomedical Sciences, Western College of Veterinary Medicine, University of Saskatchewan, 52 Campus Drive, Saskatoon, Saskatchewan S7N 5B4, Canada.; 4 Department for Innovation in Biological, Agro-food and Forest Systems (DIBAF), University of Tuscia, Viterbo, Italy

## Abstract

The gut microbiota regulates metabolic pathways that modulate the physiological state of hunger or satiety. Nutrients in the gut stimulate the release of several appetite modulators acting at central and peripheral levels to mediate appetite and glucose metabolism. After an eight-day exposure of zebrafish larvae to probiotic *Lactobacillus rhamnosus*, high-throughput sequence analysis evidenced the ability of the probiotic to modulate the microbial composition of the gastrointestinal tract. These changes were associated with a down-regulation and up-regulation of larval orexigenic and anorexigenic genes, respectively, an up-regulation of genes related to glucose level reduction and concomitantly reduced appetite and body glucose level. BODIPY-FL-pentanoic-acid staining revealed higher short chain fatty acids levels in the intestine of treated larvae. These results underline the capability of the probiotic to modulate the gut microbiota community and provides insight into how the probiotic interacts to regulate a novel gene network involved in glucose metabolism and appetite control, suggesting a possible role for *L. rhamnosus* in the treatment of impaired glucose tolerance and food intake disorders by gut microbiota manipulation.

The gut microbiota play an important role in influencing host metabolism, physiology and nutrition, lipid metabolism and growth performance^1^,^2^. Disruptions in the composition of gut microbial communities and altered interactions between microbiota-host have been linked to several intestinal diseases, including cancer and metabolic disorders^3^,^4^. Research has focused on treatments to manipulate and restore the diversity of the gut microbiota and it have been observed that probiotics modulate the microbial composition, and can also modify host nutrient metabolism and energy homeostasis^5^. The mechanisms by which the microbiota influence host metabolism are partly described.

A recent study on Non-Obese Diabetic (NOD) mice showed that changes to the gut microbiota community and metabolism which contributes to changes in the host glucose metabolism and leads to the pathogenesis of type 1 diabetes^6^. Using the *Drosophila melanogaster* model, it has been demonstrated that host genotype and microbiota are stricly correlated, with the microbiota regulating host signaling and regulatory networks^7^, including the regulation of triglycerides and glucose metabolism^8^. Using the zebrafish model, we have demonstrated that manipulation of the gut microbiota with probiotics can regulate host lipid metabolism through down-regulation of genes involved in cholesterol and triglycerides metabolism (*fit2, agpat4, dgat2, mgll, hnf4a, scap*, and *cck*)^2^. In contrast, the effect of probiotics on host glucose metabolism and appetite remains largely unkown.

In both mammalian and teleost species, appetite and glucose metabolism are controlled by complex metabolic pathways through the production of several major hormones^9^. The hypothalamic circuit has a crucial role in regulating appetite control and glucose metabolism, but peripheral organs are also responsible for the production of circulating factors that influence appetite control and glucose metabolism^10^,^11^.

A wide network of molecules regulate appetite control and among these, the hormone leptin is responsible for regulating food intake and energy expenditure^12^. Leptin was first discovered in mice and is a 16 kDa circulating hormone produced by the *obese* (*ob*) gene^13^. Leptin is mainly produced by adipose tissue; its receptors are located in the central nervous system and other tissues, such as the brain, stomach, placenta and pituitary gland^14^,^15^. Several studies have demonstrated that mutations in the *ob* gene cause leptin deficiency and leads to obesity^16^. Different studies, both in mammal and fish, reveal that leptin is able to reduce food intake by up-regulating anorexigenic signals, such as melanocortin-4-receptor (MC4R), and down-regulating orexigenic signals, such as neuropeptide Y (NPY) and cannabinoid receptor 1 (CB1)^17^,^18^. MC4R is expressed in the hypothalamic nuclei^19^ and a recent study in mice revealed that knock-out of MC4R directly induces lipid uptake and triglyceride synthesis promoting obesity^20^, meanwhile studies in humans and mice demonstrate that the activation of MC4R limits the accumulation of body fat by reducing food intake^21^.

Conversely, CB1 is mainly expressed in the brain and is responsible for food intake and weight gain^22^. It has been demonstrated that CB1 receptor increases hepatic lipid accumulation through the stimulation of the lipogenic transcription factor SREBP-1c and increases *de novo* fatty acid synthesis^23^. Like CB1, NPY and its Y receptors also stimulate food intake^24^. In the rat model, NPY has acute effects on glucose metabolism by increasing glucagon, insulin, and corticosterone^25^; moreover, a recent study demonstrated the role of NPY in stimulating lipid accumulation through pre-adipocyte proliferation, suggesting an important emergent role of NPY on adipogenesis^26^.

Previously, Oh-I^27^ and collaborators discovered an anorexigenic hormone named nesfatin-1 (*n*ucleobindin2 (NUCB2)-*e*ncoded *s*atiety- and *fat*-*in*fluencing protein) which is an 82-amino-acid peptide encoded in the N-terminal region of the precursor peptide nucleobindin 2 (NUCB2). The 396-amino-acid precursor protein NUCB2 is highly conserved among rodents and humans as well as non-mammalian vertebrate species^28^,^29^. NUCB2/Nesfatin-1 has a negative role in appetite control and inhibits NPY transcription and enhances insulin release from β-cells^27^,^30^. Among the circulating factors produced by peripheral organs, glucagon-like peptide-1 (GLP-1), a gut hormone secreted by L-cells, stimulates insulin secretions and inhibits glucagon release, promotes satiety, reduces food intake and slows gastric emptying^9^,^31^. In addition, the pancreatic endocrine tissues play a key role in the regulation of glucose metabolism through the production of several fundamental hormones such as insulin, somatostatin and glucagon, which are directly secreted into the blood^32^,^33^. When glucose is introduced with the diet, β-cells of the pancreas produce insulin to decrease the sugar level in the bloodstream, however, when there is a lack of glucose in the bloodstream, pancreatic α-cells produce glucagon which induces gluconeogenesis^9^,^33^. Moreover, ghrelin O-acyl transferase (GOAT) is highly conserved from humans to zebrafish^34^ and has been identified as the enzyme responsible for the unique n-acyl modification of ghrelin, a multifunctional orexigenic gut hormone^31^,^34^. GOAT is linked with glucose metabolism and its lack of transcription leads to increased insulin secretion and reduced body weight^35^.

The objective of the present study was to assess the effect of probiotic *Lactobacillus rhamnosus* supplementation on appetite and glucose metabolism by using *Danio rerio* larvae as a model. In order to achieve this, we assessed the zebrafish gastrointestinal (GI) microbiota after exposure to the probiotic, as well as the transcriptional regulation of genes involved in appetite control and glucose metabolism, whole organism glucose levels, and feed intake.

## Results

### *L. rhamnosus* modulates the GI microbiome

**Figure 1:**
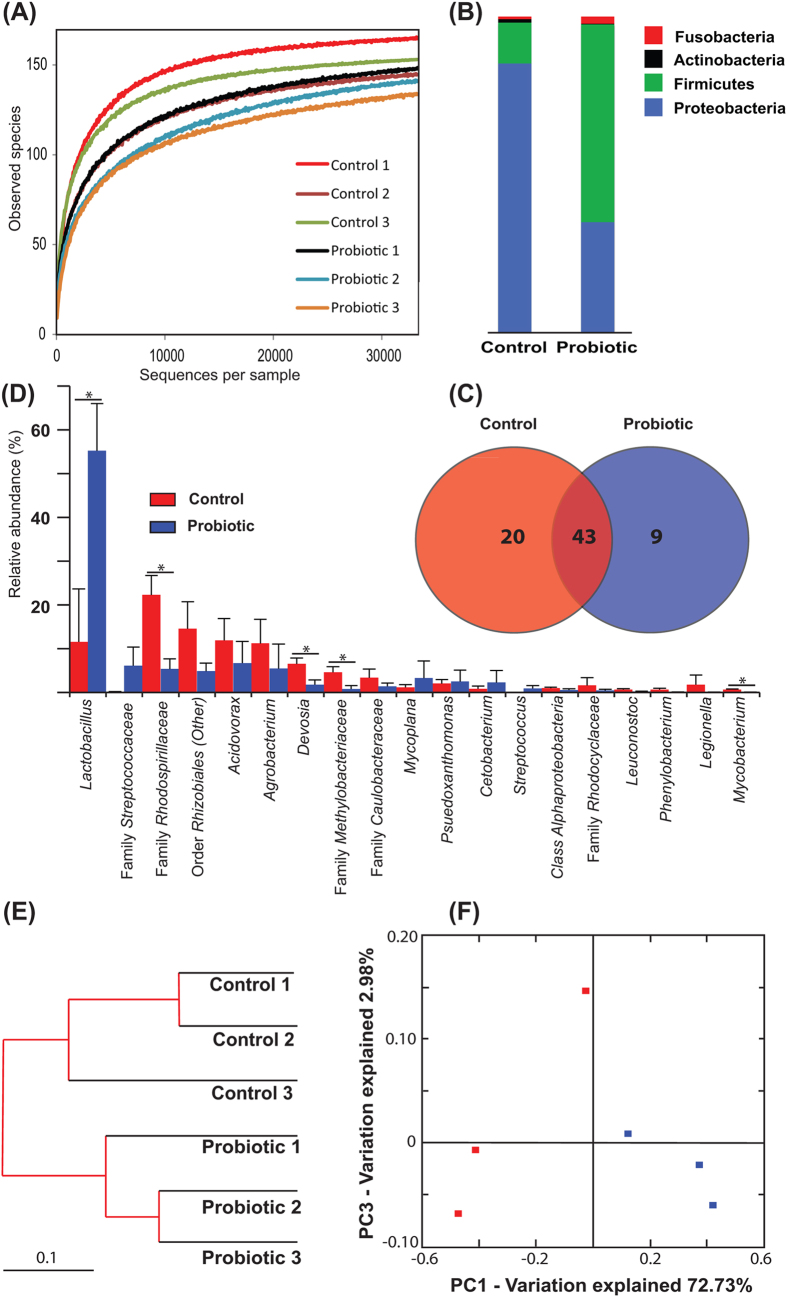
Gastrointestinal bacterial community analysis of 8 dpf zebrafish larvae. (**A**) Alpha rarefaction plot of observed species. Relative abundance of reads at the phylum (**B**) and genera (**C**) level (taxa accounting for >0.5% are represented). (**D**) Venn diagram showing the distribution of OTUs (those with >0.01% relative abundance are represented) revealing a shared community consisting of 43 OTUs. Cluster using Bray-Curtis metrics (**E**) and 2D Principal coordinate analysis (PCoA) plot of weighted UniFrac distances (**F**). *P < 0.05.

High-throughput sequence analysis of bacterial 16S rRNA V1-V2 regions at 8 days post fertilization (dpf) revealed a highly diverse microbiota with a total of 348 thousand unique reads (with an average length of 293 ± 66 bp), representing 186 OTUs, from over 1.24 million raw reads. Using USEARCH default parameters, a *de novo* UCHIME algorithm^36^ was used to identify potential chimeric sequences, which accounted for 2.5% of the total sequences. Alpha rarefaction plots of observed species reached a saturation phase at approximately 147 OTUs indicating that adequate sequence coverage was obtained to reliably describe the full diversity present in the samples (Fig. 1A). This was verified by the Good’s coverage estimation values of >99.9%, although the control group reached significantly higher coverage (0.9997 *vs* 0.9994) (Table 1). The Shannon´s diversity parameter demonstrated a significantly lower microbial diversity in the probiotic group (2.96 ± 0.59) compared to control group (4.16 ± 0.11) (*P *< 0.05) (Table 1). In order to evaluate relationships among samples based on differences in phylogenetic diversity, a dendrogram and two-dimensional principal coordinate analysis (PCoA) plot were constructed for the estimation of the dissimilarity among samples^37^ (Fig. 1E) and for the evaluation of the community composition, from weighted UniFrac distances^38^ (Fig. 1F). Both plots show clustering of the replicates from the probiotic treatment away from the control replicates, and they presented significant differences in Moran’s index, as well as the ANOSIM of Bray-Curtis similarity (*P *< 0.05), suggesting that the probiotic modified the bacterial communities in a characteristic direction.

In general, the distinguishable separation of bacterial communities found in PCoA was accompanied with significant differences in the phylogenetic composition of the gut microbiota. The bacterial communities in both treatments were dominated by two phyla: Proteobacteria and Firmicutes (Fig. 1B), however, the control group consisted of a significantly higher relative abundance of sequences belonging to the Proteobacteria (84.9%) than the probiotic group (34.8%) (*P *= 0.011), and the probiotic group showed higher relative abundance of Firmicutes (62.6%) sequences than the control group (12.9%) (*P *= 0.013). The relative abundance of Actinobacteria was significantly lower (*P *= 0.011) in the probiotic group (0.2%) than the control (1.1%). A small proportion of the reads were identified as belonging to the Cyanobacteria (0.3% in the control and 0.1% in the probiotic treatment) and Fusobacteria (0.8% in the control and 2.3% in the probiotic treatment) phyla. Taking into account the relative abundance of OTUs accounting for greater than 0.5% of the reads, 92% of the reads were resolved to order-level, 88% at family-level and 63% at genus-level (Fig. 1C). Firmicutes were dominated by the family Lactobacillaceae which was significantly higher in the treated group (*P *= 0.019). In the control group, Proteobacteria was dominated by the Families Rhodospirillaceae, Hyphomicrobiaceae, Methylobacteriaceae and Mycobacteriaceae, all of which were significantly more abundant in the control group than the probiotic (*P *< 0.05). Reads assigned to the *Lactobacillus* genus were significantly higher in the probiotic treated larvae (accounting for 55.2% of the total reads) than the control larvae (accounting for 11.5% of the reads) (*P *= 0.019); BLAST results confirmed that the lactobacilli in the probiotic samples were *L. rhamnosus* (NR-102778). Although numerically different (7.0% in the probiotic *vs* 0.2% in the control) the abundance of reads assigned to the Streptococcaceae family did not differ significantly between treatments; BLAST revealed that this genus was comprised solely by *Streptococcus thermophilus* (NR- 074827). A core microbiome, detected in both treatment groups, was identified and comprised of 43 genera (Fig. 1D); these were *Acinetobacter, Acidovorax, Agrobacterium, Bacillus, Cetobacterium, Corynebacterium, Delftia, Devosia, Hydrogenophaga, Lactobacillus, Legionella, Leuconostoc, Mycobacterium, Mycoplana, Nevskia, Paracoccus, Phenylobacterium, Plesiomonas, Pseudomonas, Pseudoxanthomonas, Propionibacterium, Rhodoplanes, Sphingopyxis, Staphylococcus, Streptococcus* genera and 18 additional genera which could not be accurately identified. Nine genera were unique to the fish treated with the probiotic (*Comamonas, Vibrio, Mesorhizobium, Sphingobium, Rubrivivax, Prosthecobacter*, one unidentified genera from the Family Pseudomonadaceae and two unidentified genera from the Orders Sphingomonadales and 34P16).Twenty genera were uniquely detected in the control fish (*Acidaminococcus, Enhydrobacter, Enterococcus, Cupriavidus, Finegoldia, Flectobacillus, Kaistobacter, Methylibium, Pediococcus, Peptoniphilus, Polynucleobacter, Trabulsiella, Weissella* and seven unidentified genera) (Fig. 1D). Additionally, a higher proportion of reads were assigned to the *Mycobacterium* genus in the control group (*P *= 0.026) (Fig. 1C).

### Probiotic treatment modulates the expression of genes involved in glucose metabolism and reduces the total glucose levels in zebrafish larvae

**Figure 2:**
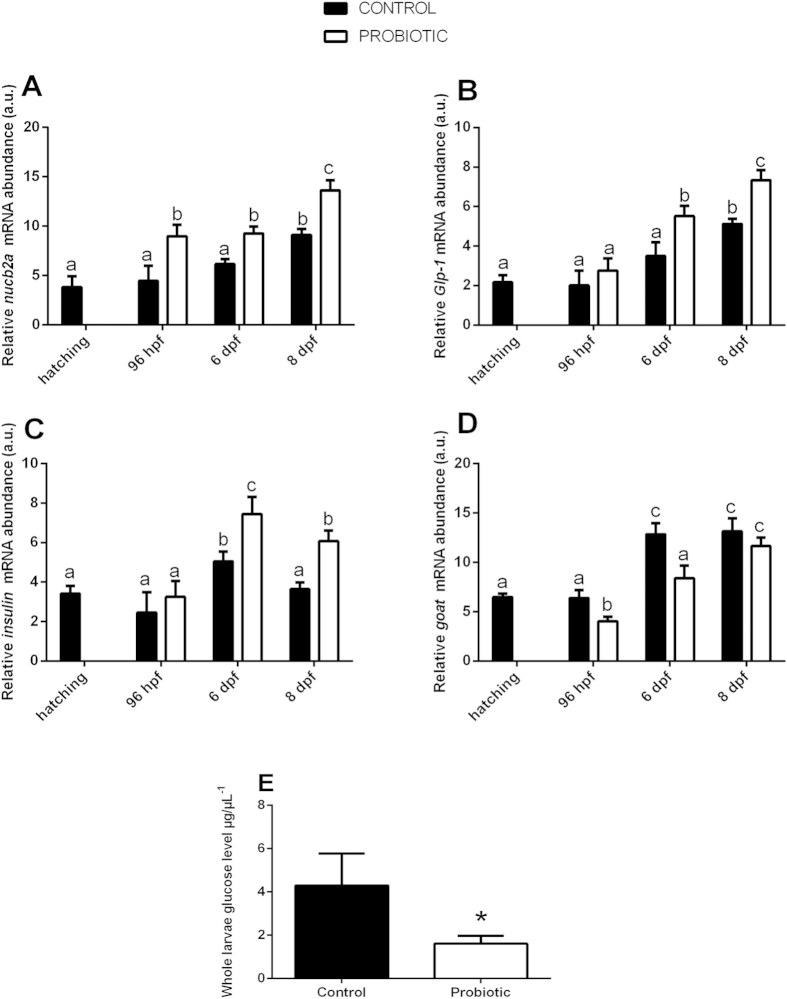
Probiotic treatment modulates the expression of genes involved in glucose metabolism and reduces the whole organism glucose levels in zebrafish larvae. Relative *nucb2a* (**A**), *Glp-1* (**B**), *insulin* (**C**), *goat* (**D**) gene expression normalized against *β-act* and *rplp,* in pools of 15 zebrafish larvae from control and probiotic groups collected at hatching, 96 hpf, 6 dpf and 8 dpf. Assays were performed in triplicate. (**E**) Total larvae glucose levels, determined from 4 pools of larvae per treatment at 8 dpf, were significantly reduced by probiotic treatment. Value with different letters and asterisk is significantly different (*P *< 0.05).

Since after the probiotic supplementation the zebrafish larvae showed modulated GI microbiota communities we wanted investigate the effects of the changes on the expression of genes involved in glucose metabolic pathways by performing Real Time PCR and glucose level analysis. *Nucb2a, glp-1* and *insulin* gene products play crucial roles in glucose metabolism through the regulation of glucose levels in the blood^9^,^27^. In particular, *nucb2a* and *Glp-1* act by up-regulating *insulin* release, the product of which decreases the sugar level in the bloodstream^30^,^33^. The results obtained by Real Time PCR revealed that the probiotic administration was able to significantly increase the transcript levels of *nucb2a* at all developmental stages (Fig. 2A) and *Glp-1* (Fig. 2B) and *insulin* at 6 dpf and 8 dpf (Fig. 2C). GOAT plays the opposite role in glucose metabolism. Energy balance directly affects *goat* mRNA expression with a down-regulation taking place in times of energy abundance which leads to an increase of insulin secretion^34^,^39^. The microbiota changes induced by probiotic treatment reduced the expression of *goat* at 96 hpf and 6 dpf compared to the control (Fig. 2D). Since the probiotic supplementation down-regulated the expression of genes that decrease glucose levels in the blood, we wanted to gain further information by measuring the glucose levels of 8 dpf whole-larva using an enzymatic assay that detects free glucose. Interestingly, the results obtained showed a significantly lower glucose level in larvae treated with probiotic (1.61 ± 0.36 μgμL^−1^) than the control larvae (4.30 ± 1.48 μgμL^−1^) (*P *= 0.04) (Fig. 2E).

### Probiotic treatment modulates the expression of genes involved in appetite control and decreases appetite in zebrafish larvae

Given that the modulation of microbiota community in the zebrafish GI after probiotic supplementation modulated transcripts involved in glucose metabolic pathways we wanted to further investigate the effects of the microbiota changes on the expression of genes related with the appetite by performing Real Time PCR and feed intake analyses.

**Figure 3:**
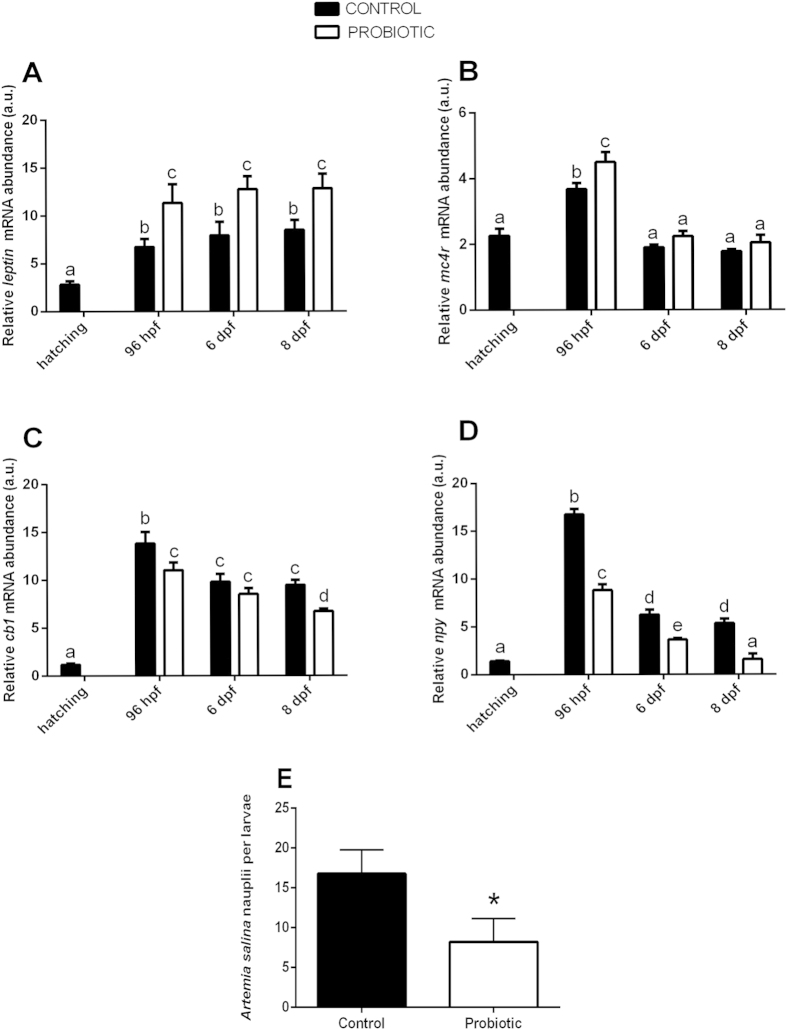
Probiotic treatment modulates the expression of genes involved in appetite control and decreases appetite in zebrafish larvae. Relative *leptin* (**A**), *mc4r* (**B**), *cb1* (**C**), *npy* (**D**) gene expression normalized against *β-act* and *rplp,* in pools of 15 zebrafish larvae from control and probiotic groups collected at hatching, 96 hpf, 6 dpf and 8 dpf. (**E**) Control and probiotic treated zebrafish larvae were fed at 8 dpf with newly-hatched *Artemia salina* nauplii. The probiotic group show significant reduction of feed intake with respect to the control. Value with different letter and asterisk is significantly different (*P *< 0.05).

*Leptin* and *mc4r* gene products are responsible for reducing food intake^15^,^40^. Our data show that the transcriptional levels of *leptin* and *mc4r* were significantly increased by the probiotic treatment. *Leptin* expression was significantly increased in the treated group in all of the developmental stages analyzed (96 hpf, 6 dpf and 8 dpf) (Fig. 3A), whereas *mc4r* expression was significantly higher only at 96 hpf (Fig. 3B).

On the contrary, *cb1* and *npy* are responsible for enhancing appetite stimulus^41^,^42^. The probiotic treatment significantly decreased the expression levels of *cb1* and *npy* transcripts, showing the same trend of expression for both genes. Compared to the control, probiotic treatment significantly down-regulated the expression of *cb1* at 96 hpf and 8 dpf, (Fig. 3C) and *npy* gene expression was significantly lower in all the developmental stages analyzed (Fig. 3D).

Since we reported a decrease of expression of orexigenic genes and an increase of anorexigenic genes, we wanted to elucidate the effect of the supplementation of probiotic *L. rhamnosus* on zebrafish appetite by measuring feed intake. At 8 dpf zebrafish larvae were fed newly-hatched *Artemia salina* nauplii and the results demonstrated a significant reduction of feed intake (8.00 ± 2.91 *A. salina* nauplii per larvae) in the probiotic treated larvae with respect to the control (17.00 ± 2.99 *A. salina* nauplii per larvae) (*P *= 0.0005) (Fig. 3E).

### Ultrastructural analysis of zebrafish gut exposed to *L. rhamnosus* evidenced an increase of absorptive surface area

**Figure 4:**
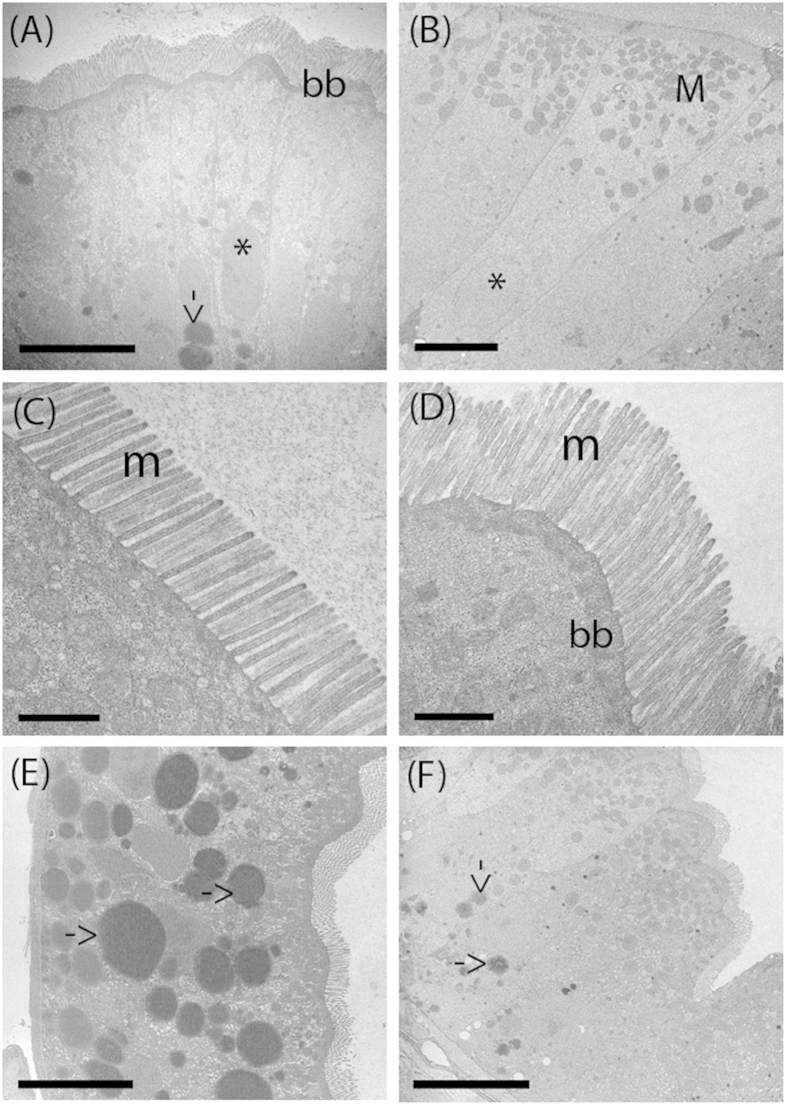
Transmission Electron Microscopy (TEM) shows the ultrastructure of the intestinal in control and probiotic treated zebrafish. Thin sections of 8 dpf zebrafish showing columnar epithelia with apical brush border in control (**A**) and probiotic treated intestine (**B**). The treated intestines present abundant spherical mitochondria located in the apical part of the enterocytes, close to the brush border. Both control and treated group larvae intestine show undamaged epithelial barrier and absence of cell debris. Electron micrographs show organized microvilli on the apical surface in the enterocyte of control (**C**) and treated larvae (**D**) and the presence of lipid droplets in the enterocytes cytoplasm of control (**E**) and treated larvae (**F**). BB: brush border; L: lumen; M: mitochondria; m: microvilli, arrow: lipid droplets; *nucleus. Scale bar: 10 μm in (**A**); 5 μm in (**B, E, F**); 1 μm in (**C**,**D**).

The proximal intestines of both control and treated larvae showed an undamaged epithelial barrier, no signs of degradation and an absence of cell debris in the lumen at 8 dpf (Fig. 4). The enterocytes lengths in the intestine of the probiotic treated larvae were significantly higher (42.54 ± 1.48 μm) compared to those of the control group (34.92 ± 2.34 μm) (*P *= 0.0001). In addition, we found significantly longer microvilli in the intestine of *L. rhamnosus* exposed larvae (1.02 ± 0.08 μm) compared to the control larvae (0.91 ± 0.07 μm) (*P  *= 0.008). Finally, TEM micrographs showed the presence of lipid droplets located in the basal enterocytes cytoplasm of both control and treated group. Probiotic treated larvae displayed significant smaller lipid droplets diameters (2.29 ± 0.37) compared to those present in the intestine epithelium of control group (3.54 ± 0.61) (*P *= 0.0001). (Table 2).

### *In vivo* short chain fatty acids localization revealed accumulation in the intestine and gallbladder of probiotic treated zebrafish

**Figure 5:**
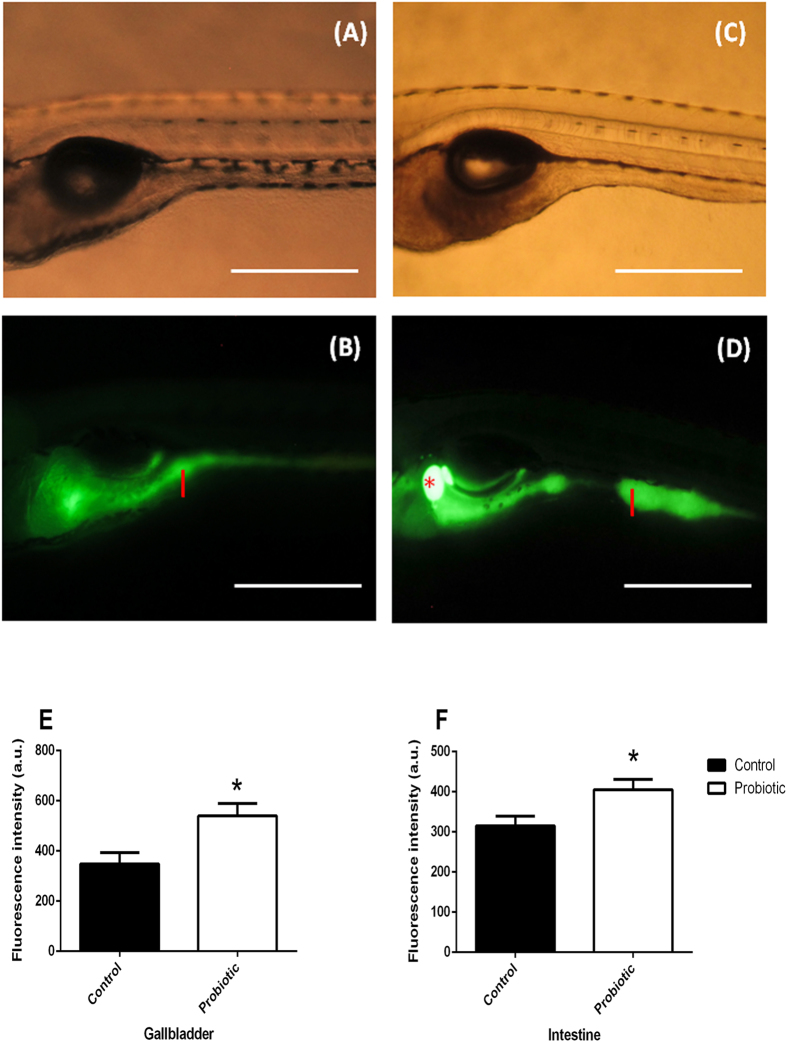
BODIPY C5 staining shows an accumulation of short chain fatty acids (SCFA) in the intestine and gallbladder of probiotic treated larvae. Representative fluorescent images of live 8 dpf zebrafish immersed in BODIPY C5 for 1 hour. Images revealed a remarkable presence of SCFA in probiotic treated larvae (**B**–**D**) that exhibited higher fluorescent signal in the intestine and gallbladder compared to the control one (**A**–**C**). Red asterisk: gallbladder; I: intestine. Scale bar: 500 μm. Probiotic treated larvae display significantly enhancement of fluorescent signal in the gallbladder (**E**) and in the intestine (**F**) with respect to the control group, highlighting an accumulation of short chain fatty acids (SCFA). Quantification of fluorescence is expressed in a.u. The data are reported as mean ± s.d from 3 individual experiments. The significance of differences between groups for these values was determined using Student’s t-test. Data are the mean ± s.d. Asterisks indicates significant differences (*P* < 0.05).

Fermentation of carbohydrates by the gut microbiota results in the production of short-chain fatty acids (SCFAs), principally propionic, acetic and butyric acids which stimulate the growth of colonic epithelial cells and confers protection to the host against infection by pathogens^43^,^44^. Since we evidenced an expansion of intestine epithelium structure (*i.e.* microvilli and enterocytes height) and changes in the gut microbiota composition, possibly stimulated by the presence of SCFAs as final products of microbiota activity, we wanted to determine whether the microbiota changes modulated the SCFAs content in the larval gut. We used BODIPY C5 (borondipyrromethene fluorescent moiety) as non-invasive *in vivo* method in order to visualize SCFA (C-5) dynamics in living tissues by fluorescence microscopy. Images showed no signs of degradation in both bodies of control (Fig. 5A) and treated larvae (Fig. 5C) after being soaked in the BODIPY C5. In addition, images obtained by fluorescence microscopy revealed significantly higher fluorescent signals in the probiotic treated gut and gallbladder (Fig. 5D) (539.7 ± 49.7 a.u., Fig. 5E) and intestine (Fig. 5D) (405.0 ± 26.0 a.u., Fig. 5F) with respect to the control gallbladder (Fig. 5B) (348.3 ± 45.0 a.u., Fig. 5E) and intestine (Fig. 5B), (315.3 ± 24.1 a.u., Fig. 5F) and suggesting an accumulation of SCFAs (*P *= 0.04).

## Discussion

In the present study we observed changes of zebrafish gut microbiota composition induced by *L. rhamnosus* administration. The resultant microbiome decreased appetite and glucose by regulating the transcription of genes involved in the control of feed intake and glucose metabolism.

As already demonstrated in previous studies in our lab, *L. rhamnosus* was able to populate the zebrafish GI tract and induce changes to the microbial composition^2^,^45^. Probiotic administration resulted in a significant increase in the proportion of reads assigned to the Firmicutes and a decrease in reads assigned to the Proteobacteria, compared to the control larvae. This was primarily caused by a significant elevation of abundance in reads assigned to the *Lactobacillus* genus (namely *L. rhamnosus*) and a substantial increase in reads assigned to the *Streptococcus* genera (namely *S. thermophilus*). This supports the observations previously demonstrated by our lab and suggests that the application of this probiotic consistently enhances the presence of *S. thermophilus* in multiple experiments at multiple zebrafish life stages (6 months old,^45^; 6 dpf,^2^; 8 dpf, this study). Elevations in the presence of these organisms coincided with relative reductions in the abundance of a number of genera, such as *Mycobacterium*, which include pathogenic species, as was previously reported in 6 dpf zebrafish larvae treated with this probiotic^2^. The significant reductions in number of species led to a reduction in the diversity of the microbiota present in the GI tract of the probiotic treated larvae.

The supplementation of *L. rhamnosus,* and the associated microbial changes in the GI tract, modulated the expression of a complex network of genes involved in appetite control and glucose metabolism. Leptin has a key role in the regulation of energy homeostasis and appetite in both fish and mammals^15^,^46^. Several studies highlighted that leptin, through the up-regulation of *mc4r* and the down-regulation of *npy* and *cb1*, is able to reduce food intake^17^,^18^,^41^. Our results evidenced that the expression of the *leptin* gene was increased by microbiota changes related with *L. rhamnosus* treatment. The up-regulation of *leptin* reflected the increase of expression of anorexigenic (*mc4r)* and a decrease of orexigenic (*npy* and *cb1*) signals in treated larvae. These results suggest a potential role of the probiotic modified microbiota to modulate these interconnected genes involved in the regulation of appetite, which was supported by the decrease of food (i.e. *Artemia salina* nauplii) intake in the probiotic treated zebrafish larvae.

The changed microbiota also affected glucose metabolism; NUCB2a/*Nesfatin-*1 gene expression was significantly up-regulated by the probiotic treatment, concomitantly with a significant increase of the expression of *insulin* gene. A number of studies have evidenced that NUCB2a/Nesftain-1, an emerging anorexigenic hormone, has a significant role in appetite control and glucose metabolism since it is able to increase insulin release from β-cells^27^,^30^,^47^. In addition, we observed that the increase of *insulin* gene expression in probiotic treated fish reflected a decrease of *goat* transcripts in the probiotic treated larvae. GOAT has emerged as molecule of interest since has been recently identified as the enzyme responsible for the activation of ghrelin, a multifunctional metabolic hormone which stimulates food intake and, moreover, maintains glucose homeostasis^39^. Studies on mice have demonstrated that the administration of GO-CoA-Tat, a bisubstrate analogue that operates as a GOAT inhibitor, caused a significant increase of insulin secretion in pancreatic β cells, and in addition, a study showed that GOAT knockout mice gained significantly less weight and had reduced fat mass when fed on a high-fat diet^35^,^48^,^49^.

These data evidenced the novel effect of the probiotic modified microbiota to acts at a transcriptional level and up-regulated the expression of genes which reduce glucose levels and concomitantly significantly reduced the glucose level in the treated larvae.

In addition, the metabolic activity of lactic acid bacteria, such as *L. rhamnosus* and *S. thermophilus*, can produce several types of SCFAs such as acetic, butyric, propionic acid and lactic acid as the end products of carbohydrate fermentation^43^,^50^. Interestingly, BODIPY C5 accumulation in the intestine and gallbladder of probiotic treated larvae indicates that this fatty acid analogue stain is absorbed by the intestine and binds FAs^51^,^52^. The enhancement of the fluorescence in the intestine of probiotic treated larvae is likely due to elevated production of SCFAs, which is indicative of increased abundance and/or activity of fermentative bacterial species. Recent studies evidenced that the SCFA butyrate enhanced the release of the peptide GLP-1, which is involved in the regulation of appetite and food intake and glucose metabolism through the up-regulation of insulin gene expression from intestinal L-cells^53^,^54^. Interestingly, our data show an up-regulation of *glp-1* gene expression level in the larvae treated with probiotic, which could be due to the production of SFCA as results of the metabolic activity of lactic acid bacteria.

We also observed that the administration of probiotic affected the architecture of the zebrafish intestine. The enterocytes in the posterior intestines of the probiotic treated larvae contained smaller lipid droplets than the control larvae. This may be due to decreases of fat storage-inducing transmembrane proteins 2 (FIT-2) transcripts as we observed in our previous work^2^ since FIT2 is implicated in the formation of lipid droplets^55^. Microvilli and enterocytes heights increased with probiotic treatment, and the improvements registered here at 8 dpf were greater than those we reported previously at 6 dpf 2. The epithelial architecture is directly correlated with the gut function and health of the host; in fact, increasing microvilli and enterocyte heights provides increased absorptive surface area. Semova and collaborators^56^ identified that the colonization of the zebrafish gut by Firmicutes promotes epithelial absorption, by modulating the energy balance of zebrafish larvae. Since, SCFAs derived from gut microbiota fermentation activities stimulate the growth of intestinal epithelial cells, we speculate that the expansion of microvilli and enterocytes in the gut of probiotic treated larvae may be due to the increased presence of SFCAs in tandem with the elevated abundance of Firmicutes^43^.

The findings we report provide novel insights on the role exerted by a *L. rhamnosus* induced modulated GI microbiome on host appetite and glucose metabolism, highlighting the hypoglicemic properties achieved by inducing transcriptional changes of a novel gene network.

The zebrafish is one of the most widely used animal models for developmental research and it is now gaining popularity and becoming an attractive model for toxicological screening and drug discovery, as well as for the study of metabolic disorders. Therefore, the results discussed here suggest a suitable potential use for *L. rhamnosus* for improving glucidic profile in impaired glucose tolerance diseases and to reduce appetite in food intake disorders.

## Material and Methods

### Animals and probiotic administration

Adult female and male zebrafish (*D. rerio*) were purchased from Acquario di Bologna (Italy) and acclimatized to the laboratory conditions (27.0 ± 0.5 °C under a 12:12 h light:dark photoperiod). Pairs were spawned individually and larvae were raised under a 12:12 h light:dark cycle at 27 °C. Embryos were collected and after hatching were divided into a control group and a probiotic-treated group. Larvae were fed a commercial diet (JBL flakes, Germany) consisting of 43.0% crude protein, 8.3% crude fat, 8.1% ash, 1.9% fibre and 8.0% moisture content. The probiotic treatment consisted of the administration of *L. rhamnosus* IMC 501® (C025396A; Synbiotec, Camerino, Italy) via the rearing water at a concentration of 10^6^ colony-forming units (CFU) ml^-1^ according to previous studies^2^. The experiment was set up in triplicates, with three control tanks and three probiotic tanks and from each tank a pool of larvae was collected at each time point. The experiment was repeated three times.

At hatching, 96 hpf, 6 dpf and 8 dpf, larvae were anesthetized using MS222 (100 mg L^−1^) (Sigma-Aldrich, Milano, Italy) and samples were collected and stored at −80 °C for Real time PCR analyses. Since at 8 dpf, molecular analysis revealed significant changes of expression of most of the genes analyzed, high through-put sequence analysis, measurement of glucose level, feed intake, Transmission Electron Microscopy (TEM) and BODIPY staining were performed at this stage of development.

All the procedures involving animals were conducted in accordance with the Italian law on animal experimentation and were approved by the Ethics Committee of Università Politecnica delle Marche (Prot #63/INT/CESA12-16). All efforts were made to minimize suffering and a humane endpoint was applied with an excess of anesthetic (MS222, Sigma- Aldrich, Milano, Italy) when animals reached a moribund state.

### DNA extraction and PCR

Larvae from 8 dpf were surface sterilized with 0.1% NaOCl for 30 s and then washed 3 times with Tris-EDTA buffer. DNA was extracted following^57^

PCR amplification of the 16S rRNA V1-V2 regions was conducted as described by^58^. One μL of DNA template was used in the PCR reactions. The PCRs was performed in a TC-512 thermal cycler (Techne, Staffordshire, UK) under the following conditions: initial denaturation at 94 °C for 7 min, then 10 cycles at 94 °C for 30 s, followed by a touchdown of 1 °C per cycle from 62 −53 °C for 30 s and 72 °C for 30 s. A further 20 cycles were performed at 94 °C for 30 s, 53 °C for 30 s, 72 °C for 30 s and a final extension 72 °C for 7 min.

### High-throughput sequence analysis

PCR products from 8 dpf were sequenced using a 318^TM^ chip (LieTechnologies^TM^) on an Ion Torrent Personal Genome Machine (LifeTechnologies^TM^) at the Systems Biology Centre in Plymouth University (UK) as described by^57^. Sequences were binned by sample and filtered within the PGM software to remove low quality reads. Data were then exported as FastQ files. Taxonomic analyses of sequence reads were performed after the removal of low quality scores (Q score <20 at 80% probability) with FASTX-Toolkit (Hannon Lab, USA). Sequences were concatenated and sorted by sequence similarity into a single fasta file. Sequences were denoised and analyzed with QIIME^59^. Briefly, OTU mapping was performed using the USEARH quality filter pipeline^60^, to remove putatively erroneous reads (chimeras), then OTU picking was achieved with a minimum pairwise identity of 97%. The most abundant sequence in each OTU were selected to assign a taxonomic classification based on the Greengenes database^61^ using the RDP classifier^62^, clustering the sequences at 97% similarity with a 0.80 confidence threshold. PyNast was used to create a multiple alignment of the representative sequences for each OTU^63^ with minimum sequence length threshold of 150 bp and 95% identification. Sequences were filtered to remove outliers, filter positions with gaps (0.95) and singletons. Highest homologous species were identified at 97% and minimum of 150 bp using nucleotide collection database at BLAST-NCBI.

Alpha diversity metrics were calculated on rarefied OTU tables with QIIME to assess sampling depth coverage using observed species, phylogenetic diversity, Chao1, Shannon’s diversity index and Good’s coverage. QIIME was also used to calculate Beta diversity metrics among samples using weighted Unifrac distances^64^ and Bray-Curtis similarity^37^. The distance matrixes were represented by a two dimensional principal coordinates analysis (PCoA) plot.

### RNA extraction and cDNA synthesis

Total RNA was extracted from 15 whole larvae per tank per time point using an RNAeasy® minikit (Qiagen, UK) following the manufacturer’s protocol. The extracted RNA was eluted following^2^.

### Real time PCR

PCRs were performed with the SYBR green method in an iQ5 iCycler thermal cycler (Bio-Rad laboratories). Triplicate PCRs were carried out for each sample analyzed following^65^.

*β-actin* (*actβ*)^66^ and *acidic ribosomal protein (rplp)*^67^ were used as the housekeeping genes to standardize the results by eliminating variation in mRNA and cDNA quantity and quality^68^. The reference genes were chosen because their mRNA levels did not vary between experimental treatments or between developmental stages. Modification of gene expression is reported with respect to the control sample. The primer sequences for *cb1, npy, leptin, mc4r, glp-1, nucb2a, rplp* and *actβ* were designed using Primer3 (210 v. 0.4.0). The primer sequences are reported in supplemental Table 1.

### Feed intake

Zebrafish larvae were maintained in tanks at 27.0 ± 0.5 °C under a 12:12 hours light:dark photoperiod. At 8 dpf, 11 zebrafish larvae per group were fed newly-hatched *Artemia salina* nauplii (approximately 400 μm) with the same concentration of *A. salina* (7 *A. salina* per μL). *A. salina* were cultured daily from cysts (*Artemia* Cysts, INVE, Thailand). Zebrafish larvae were fed for 6 min with *A. salina* nauplii, and the food intake activity of each single larvae was monitored with the aid of a Stemi 2000 micrometric Microscope (Zeiss Vision Italia, Castiglione Orona, Italy) and *A. salina* intake per larvae were counted.

### Larval glucose level

Total larvae glucose levels were determined from 4 pools of 15 larvae per treatment at 8 dpf, using an enzymatic kit that detects D-glucose (D-Fructose and D-Glucose, Megazyme, Ireland) following the manufacturer’s protocol. The concentration of each sample was determined with a spectrophotometer SHIMADZU UV-1800 (Shimadzu Scientific Instruments, USA).

The final glucose concentration was correlated to the initial pool larvae weight and finally a measurement units conversion from gL^−1^ to μgμL^−1^ and related to 1 mg of pool larvae.

### Transmission Electron Microscopy (TEM)

Samples of 10 zebrafish larvae at 8 dpf were fixed with 1% potassium dichromate, 1% osmium tetroxide and 2% glutaraldehyde in cacodylate buffer (0.1 M, pH 7.2) for 5 hours at 4 °C. Samples were processed and stained following^2^. All measurements were taken from micrographs using Software Image J^64^.

### BODIPY C-5 (3834) staining

Fifteen larvae per treatment at 8 dpf were placed into 12-well plastic dishes (3 larvae per well) and soaked in 100 μM BODIPY C-5 (4,4-Difluoro-1,3,5,7-Tetramethyl-4-Bora-3a,4a-Diaza-*s*-Indacene) (Invitrogen) diluted in 2% DMSO, then incubated for 1 hour at 28  °C in the dark. BODIPY C-5 specifically binds Short Chain Fatty Acids (SCFAs). Larvae were anaesthetized following^2^. BODIPY C-5 was excited at 505 nm (blue light) and emitted a spectrum of wavelength light which peaked at 515 nm. Regions of Interest (ROI) of 15 gallbladders and 15 intestines per group were selected. Fluorescence intensity (a.u.) was determined using Image J software^64^.

### Statistical analysis

Results were expressed as the mean ± s.d. Statistical differences were determined using 2 way ANOVA, followed by Bonferroni’s multiple comparison test. All statistical analyses were performed using Prism 6 (GraphPad Software, San Diego, CA, USA). A t-test was used to identify significant differences in feed intake, glucose levels and BODIPY fluorescence. STAMP, Venny diagram, ape and vegan packages of R were used to analyze the high-throughput sequencing data. *P*-values < 0.05 were considered significant.

## Additional Information

**How to cite this article**: Falcinelli, S. *et al.* Probiotic treatment reduces appetite and glucose level in the zebrafish model. *Sci. Rep.* **6**, 18061; doi: 10.1038/srep18061 (2016).

## Supplementary Material

Supplementary Information

## Figures and Tables

**Table 1:** Alpha diversity metrics of observed species, Chao1, Shannon’s diversity, phylogenetic diversity and Good’s coverage of zebrafish larvae of 8 dpf (mean ± s.d.; n = 3).

	Observed species	Chao1	Shannon	Phylogenetic diversity	Good’s coverage
Control	164.25 ± 7.69	174.14 ± 6.37	4.68 ± 0.21^b^	4.52 ± 0.15	0.9997 ± 0.0000^b^
Probiotic	155.09 ± 5.81	167.23 ± 3.52	3.23 ± 0.65^a^	4.30 ± 0.22	0.9995 ± 0.0001^a^

Values with different superscripts, within the same row, are significantly different at *P* < 0.05.

**Table 2:** Probiotic treatment enhances enterocytes and microvilli length and reduce lipid droplets size.

	Control	Probiotic	*P* value
Enterocytes length	34.92 ± 2.34 μm	42.54 ± 1.48 μm	0.0001
Microvilli length	0.91 ± 0.07 μm	1.02 ± 0.08 μm	0.008
Lipid droplets diameter	4.75 ± 1.12 μm	2.02 ± 0.72 μm	0.0001

Enterocytes, microvilli and lipid droplets diameter were measured in 8 dpf zebrafish larvae from control and treated group at 8 dpf.

## References

[b1] Cani P. D. & Delzenne N. M. The role of the gut microbiota in energy metabolism and metabolic disease. Curr. Pharm. Des. 15, 1546–1558 (2009). 19442172 10.2174/138161209788168164

[b2] Falcinelli S. *et al.* *Lactobacillus rhamnosus* lowers zebrafish lipid content by changing gut microbiota and host transcription of genes involved in lipid metabolism. Sci. Rep. 5 (2015). 10.1038/srep09336 PMC4378510 25822072

[b3] Guinane C. M. & Cotter P. D. Role of the gut microbiota in health and chronic gastrointestinal disease: understanding a hidden metabolic organ. Therap. Adv. Gastroenterol. 6, 295–308 (2013). 10.1177/1756283X13482996 PMC3667473 23814609

[b4] Jumpertz R. *et al.* Energy-balance studies reveal associations between gut microbes, caloric load, and nutrient absorption in humans. Am. J. Clin. Nutr. 94, 58–65 (2011). 21543530 10.3945/ajcn.110.010132 PMC3127503

[b5] Sonnenburg J. L. & Fischbach M. A. Community health care: therapeutic opportunities in the human microbiome. Sci. Transl. Med. 3, 78ps12–78ps12 (2011). 10.1126/scitranslmed.3001626 PMC3287364 21490274

[b6] Greiner T. U., Hyötyläinen T., Knip M., Bäckhed F. & Orešič M. The gut microbiota modulates glycaemic control and serum metabolite profiles in non-obese diabetic mice. PLoS ONE. 10.1371/journal.pone.0110359 (2014). PMC4229081 25390735

[b7] Dobson A. J. *et al.* Host genetic determinants of microbiota-dependent nutrition revealed by genome-wide analysis of *Drosophila melanogaster*. Nat. Commun. 10.1038/ncomms7312 (2015). PMC4333721 25692519

[b8] Chaston J. M., Newell P. D. & Douglas A. E. Metagenome-wide association of microbial determinants of host phenotype in *Drosophila melanogaster*. mBio 5, e01631–01614 (2014). 25271286 10.1128/mBio.01631-14 PMC4196228

[b9] Mojsov S. Glucagon-like peptide-1 (GLP-1) and the control of glucose metabolism in mammals and teleost fish. Am. Zool. 40, 246–258 (2000).

[b10] Karnani M. & Burdakov D. Multiple hypothalamic circuits sense and regulate glucose levels. Am. J. Physiol. Regul. Integr. Comp. Physiol. 300, R47–R55 (2011). 21048078 10.1152/ajpregu.00527.2010 PMC3023280

[b11] Dhillo W. S. Appetite regulation: an overview. Thyroid 17, 433–445 (2007). 17542673 10.1089/thy.2007.0018

[b12] Liu Q. *et al.* Expression of leptin receptor gene in developing and adult zebrafish. Gen. Comp. Endocrinol. 166, 346–355 (2010). 19941865 10.1016/j.ygcen.2009.11.015 PMC3408649

[b13] Zhang Y. *et al.* Positional cloning of the mouse obese gene and its human homologue. Nature 372, 425–432 (1994). 7984236 10.1038/372425a0

[b14] Bado A. *et al.* The stomach is a source of leptin. Nature 394, 790–793 (1998). 9723619 10.1038/29547

[b15] Johnson R. M., Johnson T. M. & Londraville R. L. Evidence for leptin expression in fishes. J. Exp. Zool. 286, 718 (2000). 10797324 10.1002/(sici)1097-010x(20000601)286:7<718::aid-jez6>3.0.co;2-i PMC3506126

[b16] Baicy K. *et al.* Leptin replacement alters brain response to food cues in genetically leptin-deficient adults. Proc. Natl. Acad. Sci. USA 104, 18276–18279 (2007). 17986612 10.1073/pnas.0706481104 PMC2084333

[b17] Muraoka O. *et al.* Leptin-induced transactivation of NPY gene promoter mediated by JAK1, JAK2 and STAT3 in the neural cell lines. Neurochem. Int. 42, 591–601 (2003). 12590942 10.1016/s0197-0186(02)00160-2

[b18] Di Marzo V. *et al.* Leptin-regulated endocannabinoids are involved in maintaining food intake. Nature 410, 822–825 (2001). 11298451 10.1038/35071088

[b19] Itoh M. *et al.* Melanocortin 4 Receptor–Deficient Mice as a Novel Mouse Model of Nonalcoholic Steatohepatitis. Am. J. Pathol. 179, 2454–2463 (2011). 21906580 10.1016/j.ajpath.2011.07.014 PMC3204024

[b20] Huszar D. *et al.* Targeted disruption of the melanocortin-4 receptor results in obesity in mice. Cell 88, 131–141 (1997). 9019399 10.1016/s0092-8674(00)81865-6

[b21] Vaisse C., Clement K., Guy-Grand B. & Froguel P. A frameshift mutation in human MC4R is associated with a dominant form of obesity. Nat. Genet. 20, 113–114 (1998). 9771699 10.1038/2407

[b22] Osei-Hyiaman D., Harvey-White J., Batkai S. & Kunos G. The role of the endocannabinoid system in the control of energy homeostasis. Int. J. Obesity 30, S33–S38 (2006). 10.1038/sj.ijo.0803276 16570103

[b23] Pai W.-Y. *et al.* Cannabinoid receptor 1 promotes hepatic lipid accumulation and lipotoxicity through the induction of SREBP-1c expression in zebrafish. Transgenic Res. 22, 823–838 (2013). 23315130 10.1007/s11248-012-9685-0

[b24] Zhang L. *et al.* Peripheral neuropeptide Y Y1 receptors regulate lipid oxidation and fat accretion. Int. J. Obesity 34, 357–373 (2009). 10.1038/ijo.2009.232 19918245

[b25] Marks J. L. & Waite K. Intracerebroventricular neuropeptide Y acutely influences glucose metabolism and insulin sensitivity in the rat. J. Neuroendocrinol. 9, 99–103 (1997). 9041362 10.1046/j.1365-2826.1997.00554.x

[b26] Rosmaninho-Salgado J. *et al.* Intracellular mechanisms coupled to NPY Y 2 and Y 5 receptor activation and lipid accumulation in murine adipocytes. Neuropeptides 46(6), 359–366 (2012). 22981159 10.1016/j.npep.2012.08.006

[b27] Oh-I S. *et al.* Identification of nesfatin-1 as a satiety molecule in the hypothalamus. Nature 443, 709–712 (2006). 17036007 10.1038/nature05162

[b28] Shimizu H. & Osaki A. Nesfatin/Nucleobindin-2 (NUCB2) and Glucose Homeostasis. Curr. Hypertens. Rev. 9(4), 270–273 (2014). 24993278

[b29] Mohan H. & Unniappan S. Phylogenetic aspects of nucleobindin-2/nesfatin-1. Curr. Pharm. Des. 19, 6929–6934 (2013). 23537083 10.2174/138161281939131127124149

[b30] Li Z., Li Y. & Zhang W. Regulation of glucose metabolism by nesfatin-1. FASEB J. 27, 1160.1162 (2013).

[b31] Lim G. E. & Brubaker P. L. Glucagon-like peptide 1 secretion by the L-cell the view from within. Diabetes 55, S70–S77 (2006).

[b32] Elo B., Villano C., Govorko D. & White L. Larval zebrafish as a model for glucose metabolism: expression of phosphoenolpyruvate carboxykinase as a marker for exposure to anti-diabetic compounds. J. Mol. Endocrinol. 38, 433–440 (2007). 17446233 10.1677/JME-06-0037

[b33] Gnügge L., Meyer D. & Driever W. Pancreas development in zebrafish. Methods Cell Biol. 76, 531–551 (2003). 15602891 10.1016/s0091-679x(04)76024-0

[b34] Yang J., Brown M. S., Liang G., Grishin N. V. & Goldstein J. L. Identification of the acyltransferase that octanoylates ghrelin, an appetite-stimulating peptide hormone. Cell 132, 387–396 (2008). 18267071 10.1016/j.cell.2008.01.017

[b35] Shlimun A. & Unniappan S. Ghrelin O-acyl transferase: bridging ghrelin and energy homeostasis. Int. J. Pept. org/10.1155/2011/217957 (2011). 10.1155/2011/217957 PMC3175403 21941572

[b36] Edgar R. C., Haas B. J., Clemente J. C., Quince C. & Knight R. UCHIME improves sensitivity and speed of chimera detection. Bioinformatics 27, 2194–2200 (2011). 21700674 10.1093/bioinformatics/btr381 PMC3150044

[b37] Bray J. R. & Curtis J. T. An ordination of the upland forest communities of southern Wisconsin. Ecol. Monogr. 27, 325–349 (1957).

[b38] Lozupone C. A., Hamady M., Kelley S. T. & Knight R. Quantitative and qualitative β diversity measures lead to different insights into factors that structure microbial communities. Appl. Environ. Microbiol. 73, 1576–1585 (2007). 17220268 10.1128/AEM.01996-06 PMC1828774

[b39] Mohan H. & Unniappan S. Discovery of ghrelin O-acyltransferase. Endocr. Dev. 25, 16–24 (2013). 23652388 10.1159/000346039

[b40] Balthasar N. *et al.* Divergence of melanocortin pathways in the control of food intake and energy expenditure. Cell 123, 493–505 (2005). 16269339 10.1016/j.cell.2005.08.035

[b41] Di Marzo V. & Matias I. Endocannabinoid control of food intake and energy balance. Nat. Neurosci. 8, 585–589 (2005). 15856067 10.1038/nn1457

[b42] Bergen H. T., Mizuno T., Taylor J. & Mobbs C. V. Resistance to diet-induced obesity is associated with increased proopiomelanocortin mRNA and decreased neuropeptide Y mRNA in the hypothalamus. Brain Res. 851, 198–203 (1999). 10642844 10.1016/s0006-8993(99)02186-1

[b43] Donohoe D. R. *et al.* The microbiome and butyrate regulate energy metabolism and autophagy in the mammalian colon. Cell metab. 13, 517–526 (2011). 21531334 10.1016/j.cmet.2011.02.018 PMC3099420

[b44] Hooper L. V., Midtvedt T. & Gordon J. I. How host-microbial interactions shape the nutrient environment of the mammalian intestine. Annu. Rev. Nutr. 22, 283–307 (2002). 12055347 10.1146/annurev.nutr.22.011602.092259

[b45] Gioacchini G. *et al.* Interplay between autophagy and apoptosis in the development of *Danio rerio* follicles and the effects of a probiotic. Reprod. Fertil. Dev. 25, 1115–1125 (2013). 23195281 10.1071/RD12187

[b46] Sobhani I. *et al.* Leptin secretion and leptin receptor in the human stomach. Gut 47, 178–183 (2000). 10896907 10.1136/gut.47.2.178 PMC1727985

[b47] Price C. J., Samson W. K. & Ferguson A. V. Nesfatin-1 inhibits NPY neurons in the arcuate nucleus. Brain Res. 1230, 99–106 (2008). 18625211 10.1016/j.brainres.2008.06.084 PMC2590930

[b48] Zhao T.-J. *et al.* Ghrelin O-acyltransferase (GOAT) is essential for growth hormone-mediated survival of calorie-restricted mice. Proc. Natl. Acad. Sci. USA 107, 7467–7472 (2010). 20231469 10.1073/pnas.1002271107 PMC2867684

[b49] Kirchner H. *et al.* GOAT links dietary lipids with the endocrine control of energy balance. Nat. Med. 15, 741–745 (2009). 19503064 10.1038/nm.1997 PMC2789701

[b50] Ogawa J. *et al.* Production of conjugated fatty acids by lactic acid bacteria. J. Biosci. Bioeng. 100, 355–364 (2005). 16310724 10.1263/jbb.100.355

[b51] Schlegel A. & Stainier D. Y. Microsomal triglyceride transfer protein is required for yolk lipid utilization and absorption of dietary lipids in zebrafish larvae. Biochemistry 45, 15179–15187 (2006). 17176039 10.1021/bi0619268

[b52] Otis J. P. & Farber S. A. Imaging vertebrate digestive function and lipid metabolism *in vivo*. Drug. Discov. Today Dis. Models 10, e11–e16 (2013). 10.1016/j.ddmod.2012.02.008 PMC3811959 24187571

[b53] Dailey M. J. & Moran T. H. Glucagon-like peptide 1 and appetite. Trends Endocrinol. Metab. 24, 85–91 (2013). 23332584 10.1016/j.tem.2012.11.008 PMC3594872

[b54] Yadav H., Lee J.-H., Lloyd J., Walter P. & Rane S. G. Beneficial metabolic effects of a probiotic via butyrate-induced GLP-1 hormone secretion. J. Biol. Chem. 288, 25088–25097 (2013). 23836895 10.1074/jbc.M113.452516 PMC3757173

[b55] Gross D. A., Zhan C. & Silver D. L. Direct binding of triglyceride to fat storage-inducing transmembrane proteins 1 and 2 is important for lipid droplet formation. Proc. Natl. Acad. Sci. USA 108, 19581–19586 (2011). 22106267 10.1073/pnas.1110817108 PMC3241795

[b56] Semova I. *et al.* Microbiota regulate intestinal absorption and metabolism of fatty acids in the zebrafish. Cell Host Microbe 12, 277–288 (2012). 22980325 10.1016/j.chom.2012.08.003 PMC3517662

[b57] Standen B. *et al.* Modulation of the intestinal microbiota and morphology of tilapia, *Oreochromis niloticus,* following the application of a multi-species probiotic. Appl. Microbiol. Biotechnol. 1–15 (2015). 10.1007/s00253-015-6702-2 26115752

[b58] Roeselers G. *et al.* Evidence for a core gut microbiota in the zebrafish. ISME J. 5, 1595–1608 (2011). 21472014 10.1038/ismej.2011.38 PMC3176511

[b59] Caporaso J. G. *et al.* QIIME allows analysis of high-throughput community sequencing data. Nat. Methods 7, 335–336 (2010). 20383131 10.1038/nmeth.f.303 PMC3156573

[b60] Edgar R. C. Search and clustering orders of magnitude faster than BLAST. Bioinformatics 26, 2460–2461 (2010). 20709691 10.1093/bioinformatics/btq461

[b61] DeSantis T. Z. *et al.* Greengenes, a chimera-checked 16S rRNA gene database and workbench compatible with ARB. Appl. Environ. Microb. 72, 5069–5072 (2006). 10.1128/AEM.03006-05 PMC1489311 16820507

[b62] Wang Q., Garrity G. M., Tiedje J. M. & Cole J. R. Naive Bayesian classifier for rapid assignment of rRNA sequences into the new bacterial taxonomy. Appl. Environ. Microb. 73, 5261–5267 (2007). 10.1128/AEM.00062-07 PMC1950982 17586664

[b63] Caporaso J. G. *et al.* PyNAST: a flexible tool for aligning sequences to a template alignment. Bioinformatics 26, 266–267 (2010). 19914921 10.1093/bioinformatics/btp636 PMC2804299

[b64] Abràmoff M. D., Magalhães P. J. & Ram S. J. Image processing with ImageJ. Biophotonics international 11, 36–42 (2004).

[b65] Maradonna F. *et al.* Probiotic Supplementation Promotes Calcification in *Danio rerio* Larvae: A Molecular Study. PloS one 8, e83155 (2013). 24358259 10.1371/journal.pone.0083155 PMC3866187

[b66] McCurley A. T. & Callard G. V. Characterization of housekeeping genes in zebrafish: male-female differences and effects of tissue type, developmental stage and chemical treatment. BMC Mol. Biol. 9, 102 (2008). 19014500 10.1186/1471-2199-9-102 PMC2588455

[b67] Aursnes I. A., Rishovd A. L., Karlsen H. E. & Gjøen T. Validation of reference genes for quantitative RT-qPCR studies of gene expression in Atlantic cod (Gadus morhua l.) during temperature stress. BMC Res. Notes 4, 104 (2011). 21466674 10.1186/1756-0500-4-104 PMC3080820

[b68] Bustin S. A. *et al.* The MIQE guidelines: minimum information for publication of quantitative real-time PCR experiments. Clin. Chem. 55, 611–622 (2009). 19246619 10.1373/clinchem.2008.112797

