# Supplementary material for: Probiotic treatment reduces appetite and glucose level in the zebrafish model
Source: Sci Rep. 2016 Jan 5;6:18061. doi: 10.1038/srep18061 (PMC4700460; doi:10.1038/srep18061)
Supplement: Supplementary Information [file srep18061-s1.pdf]

Supplemental information

Probiotic treatment reduces appetite and glucose level in the zebrafish model

Silvia Falcinelli[1], Ana Rodiles[2], Suraj Unniappan[3], Simona Picchietti[4], Giorgia Gioacchini[1], Daniel Lee Merrifield[2] and Oliana Carnevali[1]*

Table S1. Primer sequences used for real-time polymerase chain reaction analyses

Gene	Forward	Reverse
cb1	**5'- TCTGTGGGAAGCCTGTTTC -3'**	**5'- ACCGAGTTGAGCCGTTTG -3'**
npy	**5'- GTCTGCTTGGGGACTCTCAC -3'**	**5'- CGGGACTCTGTTTCACCAAT -3'**
leptin	**5'- AGCTCTCCGCTCAACCTGTA -3'**	**5'- CAGCGGGAATCTCTGGATAA -3'**
mc4r	**5'- ATCTCCACGGAGGTCTTCCT -3'**	**5'- CGAAGCATTGGAGACACTCA -3'**
glp-1	**5'- CAGAGGGAACTTTCTCCAACGA -3'**	**5'- AGTTCATACCCATTTCTCTTAGCGT -3'**
insulin	**5'- TAAGCACTAACCCAGGCACA -3'**	**5'- GATTTAGGAGGAAGGAAACC -3'**
goat	**5'- GCACACTGGAAGATGAGCGA -3'**	**5'- AACCGAAGGACGACTGGATG -3'**
nucb2a	**5'- AGGAGCGGCATGAAGAATTT -3'**	**5'- GATGGTTGACTTTGGGGTGA -3'**
rplp	**5'- CTGAACATCTCGCCCTTCTC -3'**	**5'- TAGCCGATCTGCAGACACAC -3'**
actβ	**5'- GGTACCCATCTCCTGCTCCAA -3'**	**5'- GAGCGTGGCTACTCCTTCACC -3'**

The primer sequences for, *cb1*, *npy, leptin, mc4r, glp-1, insulin, goat, nucb2a, rplp* and *actβ* were designed using Primer3 (210 v. 0.4.0).
